# Antimalarial Activity of Artefenomel Against Asexual Parasites and Transmissible Gametocytes During Experimental Blood-Stage *Plasmodium vivax* Infection

**DOI:** 10.1093/infdis/jiaa287

**Published:** 2020-06-01

**Authors:** Katharine A Collins, Azrin N Abd-Rahman, Louise Marquart, Emma Ballard, Nathalie Gobeau, Paul Griffin, Stephan Chalon, Jörg J Möhrle, James S McCarthy

**Affiliations:** 1 QIMR Berghofer Medical Research Institute, Herston Australia; 2 Medicine for Malaria Venture, Meyrin, Switzerland; 3 The University of Queensland, Brisbane, Australia; 4 Department of Medicine and Infectious Diseases, Mater Hospital and Mater Research, South Brisbane, Australia

**Keywords:** *Plasmodium vivax*, artefenomel, OZ439, Malaria, Volunteer infection studies, IBSM, transmission

## Abstract

**Background:**

Interventions that effectively target *Plasmodium vivax* are critical for the future control and elimination of malaria. We conducted a *P. vi*v*ax* volunteer infection study to characterize the antimalarial activity of artefenomel, a new drug candidate.

**Methods:**

Eight healthy, malaria-naive participants were intravenously inoculated with blood-stage *P. vivax* and subsequently received a single oral 200-mg dose of artefenomel. Blood samples were collected to monitor the development and clearance of parasitemia, and plasma artefenomel concentration. Mosquito feeding assays were conducted before artefenomel dosing to investigate parasite transmissibility.

**Results:**

Initial parasite clearance occurred in all participants after artefenomel administration (log_10_ parasite reduction ratio over 48 hours, 1.67; parasite clearance half-life, 8.67 hours). Recrudescence occurred in 7 participants 11–14 days after dosing. A minimum inhibitory concentration of 0.62 ng/mL and minimum parasiticidal concentration that achieves 90% of maximum effect of 0.83 ng/mL were estimated, and a single 300-mg dose was predicted to clear 10^9^ parasites per milliliter with 95% certainty. Gametocytemia developed in all participants and was cleared 4–8 days after dosing. At peak gametocytemia, 75% of participants were infectious to mosquitoes.

**Conclusions:**

The in vivo antimalarial activity of artefenomel supports its further clinical development as a treatment for *P. vivax* malaria.

**Clinical Trials Registration:**

NCT02573857.

Malaria is a major cause of disease and death worldwide, with an estimated 405 000 malaria-related deaths occurring in 2018 [1]. Although *Plasmodium falciparum* is responsible for the majority of deaths, *Plasmodium vivax* is the predominant human malaria parasite species outside of Africa and has become recognized as a considerable cause of severe morbidity and a contribution to mortality [2, 3]. There have been significant advances in the treatment and prevention of malaria over the past decade; these have led to a reduction in both the incidence and mortality rate of malaria in some areas [1]. However, these advances have been almost exclusively targeted toward control of *P. falciparum*, and there has been a paucity of research for *P. vivax*–specific interventions [3, 4].

Chloroquine has been the drug of choice for treatment of *P. vivax* infection after a safe and effective dosage regimen was defined in 1946 [5]. Although chloroquine has been the first-line treatment for *P. vivax* malaria, resistance is now prevalent in many endemic countries [6], and artemisinin combination therapy is now a recommended treatment [1, 7, 8]. Artemisinin-resistant *P. falciparum* is also emerging; thus, there is a clear need for the development of new antimalarials for *P. vivax* as well as *P. falciparum*. The 8-aminoquinoline tafenoquine was recently approved by the US Food and Drug Administration for the treatment of the dormant liver stage (hypnozoite) in *P. vivax* infection and for malaria chemoprophylaxis but can be used only in patients with normal glucose-6-phosphate dehydrogenase activity and is contraindicated in children, during pregnancy, and in lactating mothers with infants of deficient or unknown glucose-6-phosphate dehydrogenase status [9].

Several other new antimalarial candidates are currently under clinical development (reviewed in [10]). Although new drugs are now generally evaluated against both *P. falciparum* and *P. vivax* in field studies, they are typically only tested against *P. falciparum* during early clinical development [7]. Malaria volunteer infection studies have been shown to accelerate the evaluation of new antimalarial drugs for *P. falciparum*, shortening the time from first-in-human to the selection of an effective dose [11]. We have described the development of a safe and reproducible *P. vivax* volunteer infection study where the induced blood-stage malaria (IBSM) model is used to initiate malaria infection by intravenous injection of *P. vivax–*infected erythrocytes [12–14]. This model can be used for early clinical evaluation of experimental antimalarials against *P. vivax.*

The synthetic ozonide antimalarials artefenomel and arterolane (previously known as OZ439 and OZ277, respectively) are thought to act in a similar manner to artemisinins by reacting with ferrous iron in the parasite digestive vacuole to produce free radicals leading to alkylation of key parasitic proteins [15]. The in vitro antimalarial activity of artefenomel toward all asexual erythrocytic *P. falciparum* stages, and in vivo efficacy in *Plasmodium berghei*–infected mice, has been established [16]. In addition, the ex vivo antimalarial activity of artefenomel toward several *P. falciparum* and *P. vivax* clinical isolates has been characterized [17]. The clinical development of artefenomel has advanced to phase 2 clinical studies [18–20], and its key pharmacokinetic-pharmacodynamic (PK/PD) parameters have been defined in a volunteer infection study using the *P. falciparum* IBSM model [18]. Artefenomel has a good safety profile up to 1600 mg [19], and it exhibits good antiparasitic activity against both *P. falciparum* and *P. vivax* when administered as single oral doses of 200–1200 mg to patients with malaria [20].

In the current study, we aimed to define the PK/PD relationship of artefenomel using the *P. vivax* IBSM model and to establish the minimum dose of artefenomel required to clear *P. vivax* blood-stage parasitemia. We evaluated the safety, tolerability, PK, and PD antimalarial activity associated with a single 200-mg dose of artefenomel in the *P. vivax* IBSM model, and we also investigated the development of gametocytemia and transmissibility of gametocytes to mosquitoes.

## METHODS

### Study Design and Participants

This was a 2-part, phase 1, open-label, volunteer infection study using the IBSM model. Part 1 has been reported separately [21]. Part 2, reported here, investigated the antimalarial activity of artefenomel toward *P. vivax*. Healthy malaria-naive men and women of nonchildbearing potential, aged 18–55 years, were eligible for inclusion. A complete list of the eligibility criteria is included in the Supplementary Materials. All participants gave written informed consent. This study was approved by the QIMR Berghofer Medical Research Institute Human Research Ethics Committee, and was registered with ClinicalTrials.gov (NCT02573857).

### Procedures

A single cohort of 8 participants was inoculated intravenously on day 0 with *P. vivax–*infected erythrocytes (approximately 564 viable parasites), and parasitemia was monitored with quantitative polymerase chain reaction (qPCR) targeting the *P. vivax* 18S ribosomal RNA gene [13]. The *P. vivax* HMP013 isolate used for inoculation was collected in 2014 from a traveler returning to Australia from India who presented with malaria-related symptoms; a cryopreserved parasite bank was prepared and tested as described elsewhere [14].

A single oral 200-mg dose of artefenomel (Penn Pharmaceuticals) was administered on day 10 after an overnight fast. Artefenomel powder was suspended in 0.8% polysorbate aqueous solution (Ora-sweet) and taken orally with 200 mL of full-cream milk. The dose selected was below the estimated single dose required to achieve complete cure. Permitting recrudescence best allows for characterization of the PK/PD relationship between artefenomel plasma concentration and *P. vivax* parasitemia. Data obtained in the previous *P. falciparum* IBSM study [18], and the phase 2a monotherapy study of artefenomel in patients with *P. falciparum* or *P. vivax* malaria [20], informed dose selection.

It was planned in the study protocol that a second dose of artefenomel (400 mg) would be administered in the event of recrudescence. However, given that a number of cases of elevated liver function test enzymes were observed after administration of the first dose (see safety results section), it was decided not to administer a second dose. Instead, participants received a standard course of artemether-lumefantrine (Riamet; Novartis Pharmaceuticals) in response to recrudescence, or on day 28 if recrudescence did not occur. Gametocytemia was monitored by means of reverse-transcriptase qPCR targeting a messenger RNA marker of mature female gametocytes, *pvs25* [14]. Transcripts per milliliter were converted to gametocytes per milliliter, using the transcript number estimates per gametocyte calculated elsewhere [22]. Participants were monitored until the end of the study (days 28–46). Supplementary Table 1 summarizes the timing of study procedures.

### PK Analysis

Blood samples were collected at the following time points after dosing to determine the artefenomel plasma concentration: 1, 2, 3, 4, 6, 8, 12, 16, 24, 48, 72, and 96 hours and between 120 and 168 hours. Samples were analyzed by means of high-performance liquid chromatography–tandem mass spectrometry, as described elsewhere [19]. Noncompartmental PK analysis was performed using R software, version 3.3.2. PK end points were the maximum plasma concentration, the time point when the maximum concentration was reached, the area under the concentration-time curve up to last time point measure, the area under the concentration-time curve extrapolated to infinity, and the elimination half-life.

### PD Analysis

To monitor parasitemia by 18S qPCR, blood samples were collected daily from day 4 until positive for malaria, and twice daily until artefenomel dosing. Samples were then collected at 4, 8, 12, 16, 24, 30, 36, 48, 60, 72, 84, 96, and 108 hours after dosing; subsequent sampling was performed approximately 3 times per week. The PD variables of interest in this study were the parasite reduction ratio (PRR) and parasite clearance half-life. The PRR provides an estimate of the antimalarial activity of a compound and is the ratio of the parasite density decrease over a 48-hour period (expressed as the overall cohort-specific log_10_ PRR_48_). The log_10_ PRR_48_ was estimated using the slope of the optimal fit of the log-linear relationship of the parasitemia decay, as described elsewhere [23].

### PK/PD Modeling

PK/PD modeling included data from 2 previous IBSM studies in addition to data obtained in the current study (Supplementary Table 2). The demographic characteristics of participants included in the PK and PD data sets are summarized in Supplementary Table 3. Data preparation, exploration, model definition, model evaluation and simulations were performed using R software (version 3.4.2) and the R package IQRtools (version 0.9.1; IntiQuan) within the software package MonolixSuite 2016R1 (Lixoft). The minimum inhibitory concentration (MIC) and the minimum parasiticidal concentration that achieves 90% of maximum effect (MPC_90_) were derived from the PK/PD model. Simulations were performed to predict the minimum effective single dose, defined as the dose that clears a parasite count of 10^9^/mL.

### Safety Assessment

Safety assessments included adverse event (AE) recording, physical examinations, vital signs monitoring, electrocardiograms, clinical laboratory evaluation (hematology, biochemistry, and urinalysis), and malaria clinical score recording. The malaria clinical score served as a clinical indication of the severity of the induced malaria infection; 14 signs and symptoms commonly associated with malaria were graded on a 3-point scale (where 0 indicates absent; 1, mild; 2, moderate; and 3, severe), and the values were summed in order to generate an overall score (maximum possible score, 42). The medical assessment of AE severity was graded on a 4-point scale (mild, moderate, severe, and very severe) in accordance with the *WHO Handbook for Reporting of Results of Cancer Treatment* [24].

### Transmission to Mosquitoes

The transmission of parasites from participants to *Anopheles stephensi* mosquitoes was measured using direct skin feeding assays and direct membrane feeding assays on day 8, and before artefonomel administration on day 10. For the direct feeding assays, approximately 35 mosquitos were allowed to bite on the volar surface of the participants forearm for approximately 15 minutes. For the direct membrane feeding assays, approximately 65 mosquitoes were allowed to feed on whole blood collected into lithium heparin vacutainers, kept at ≥37°C, before transfer into glass membrane feeders where mosquito feeding was allowed for approximately 30 minutes. For both assays, mosquitoes were maintained at 27°C and 70% –80% humidity for 7–10 days, when the mosquitoes were dissected to quantify oocysts in midgut preparations using 18S qPCR [25, 26].

### Sample Size

The planned sample size of the current study (n = 8) was determined from previously published IBSM challenge studies that were sufficiently powered to obtain statistically meaningful data on the effects of experimental antimalarials on malaria parasite kinetics.

## RESULTS

### Study Participants

The study was conducted between April and June 2016. Eight participants were enrolled in the study; all were male and white (Table 1). All participants were inoculated with blood-stage *P. vivax* parasites on day 0, received a single oral dose of 200 mg artefenomel on day 10, and completed the study per protocol.

**Table 1.** Demographic Profile of Participants (N = 8)

Characteristic	Value
Age, mean (SD) [range], y	24.9 (4.6) [21–33]
Male sex, no. (%)	8 (100.0)
White race, no. (%)	8 (100.0)
BMI, mean (SD) [range]^a^	22.6 (2.8) [18.8–26.9]
Height, mean (SD) [range], cm	184.1 (5.9) [176–194]
Weight, mean (SD) [range], kg	77.0 (13.1) [58.3–96.6]

Abbreviations: BMI, body mass index; SD, standard deviation.

^a^Body mass index was calculated as weight in kilograms divided by height in meters squared.

### Safety

A total of 196 AEs were recorded; the majority were deemed related to malaria (170 of 196 [86.7%]) and were mild or moderate in severity (Table 2 and Supplementary Table 6). No serious AEs were reported and no AEs resulted in study discontinuation. Eight AEs in 3 participants were considered possibly related to artefenomel, although all of these were also considered possibly related to malaria. These AEs included 1 case each of mild vomiting, moderate fatigue, and mild myalgia and 5 cases of headache (mild or moderate).

**Table 2.** Summary of Adverse Events (N = 8)

Type of AE^a^	Participants With ≥1 AE, No. (%)	AEs, No.
Any AE	8 (100)	196
AE possibly related to artefenomel	3 (37.5)	8
Moderate AE (grade 2)	8 (100)	55
Moderate AE (grade 2) possibly related to artefenomel	2 (25)	3
Severe AE (grade 3)	8 (100)	11
Severe AE (grade 3) possibly related to artefenomel	0	0
AE leading to discontinuation of treatment	0	0
SAE	0	0

Abbreviations: AE, adverse event; SAE, serious AE.

^a^The medical assessment of AE severity was graded on a 4-point scale (1, mild; 2, moderate; 3, severe; 4, very severe), in accordance with the *WHO Handbook for Reporting of Results of Cancer Treatment* [24].

Eleven AEs were classified as severe: 4 cases of elevated alanine aminotransferase (peak, 13.4 times the upper limit of normal), 1 case of elevated aspartate aminotransferase (peak, 7.3 times the upper limit of normal), 3 cases of pyrexia (peak, 39.8°C), and 3 cases of lymphopenia (lowest lymphocyte count, 0.41 × 10^9^/L). The elevations in alanine aminotransferase and aspartate aminotransferase levels were not accompanied by an elevation in bilirubin; thus, Hy’s law was not reached. All severe AEs were considered related to malaria and resolved without treatment or with the use of standard antipyretic medication.

The severity of malaria symptoms and signs experienced by participants during the study assessed by the malaria clinical scoring tool are presented in Supplementary Table 7. At the time of artefenomel dosing, 1 participant had no malaria symptoms or signs (clinical score, 0), 4 had mild symptoms or signs (clinical score, 2 or 3) and 3 had moderate symptoms or signs (clinical score, 10–13). The malaria symptoms or signs worsened for 5 participants in the 12 hours following artefenomel administration. The peak clinical score recorded during the study was 14, which occurred in 1 participant.

### PK Parameters

**Figure 1.**
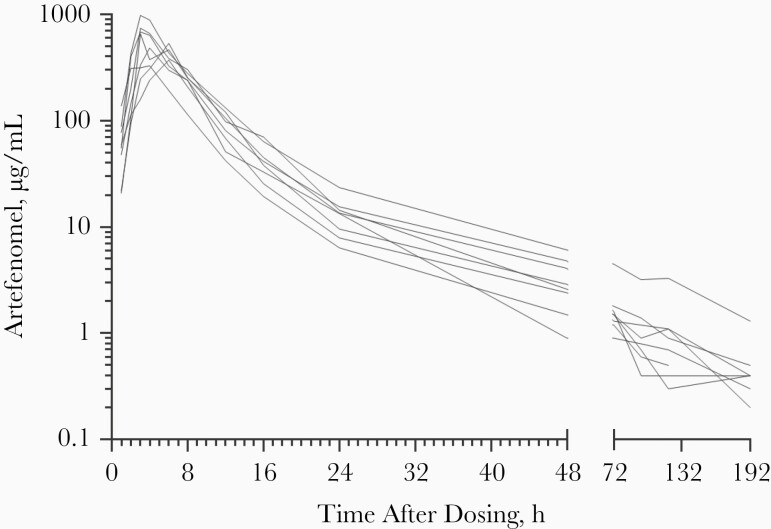
Artefenomel plasma concentration–time profiles for individual participants.

Artefenomel plasma concentration–time profiles were similar between participants (Figure 1), and variability in noncompartmental PK parameters between participants was moderate (Table 3). Absorption of artefenomel was rapid, with peak plasma concentrations occurring at a median of 3.5 hours after administration, while the elimination half-life was moderate, ranging from 37 to 87 hours.

**Table 3.** Noncompartmental Pharmacokinetic Analysis (N = 8)

Pharmacokinetic Parameter	Geometric Mean (Range)
*C*_max_, ng/mL	566 (329–981)
*t*_max_, h	3.5 (3.0–6.0)a
AUC_0–last_, h⋅ng/mL	3712 (2413–6158)
AUC_0–∞_, h⋅ng/mL	3748 (2432–6284)
*t*_½_ , h	56 (37–87)

Abbreviations: AUC_0–last_, area under the curve up to last time point measure; AUC_0–∞_, area under the curve extrapolated to infinity; *C*_max_, maximum concentration; *t*_½_ , elimination half-life; *t*_max_, time when maximum concentration is reached.

atmax values are Median and not Geometric Mean.

### PD Analysis of Parasite Clearance

**Figure 2.**
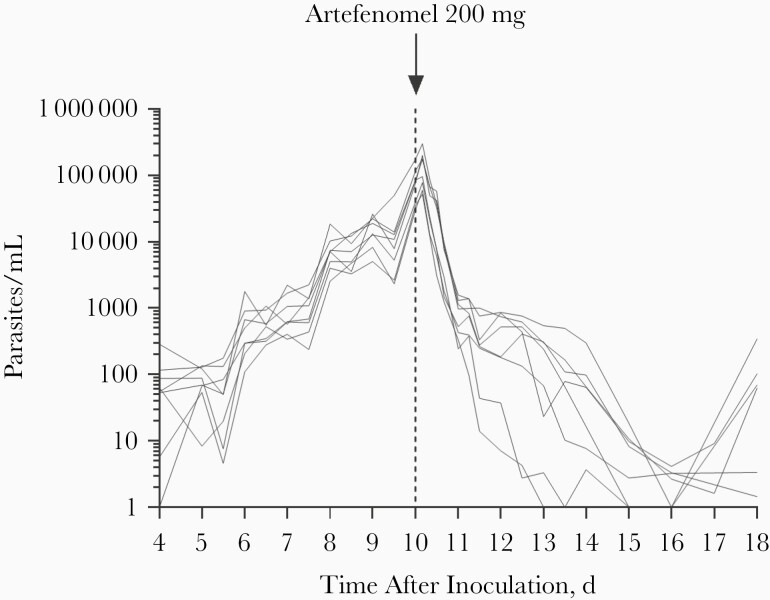
Individual participant parasitemia profiles. Participants (n = 8) were inoculated intravenously with *Plasmodium vivax*–infected erythrocytes on day 0 and were administered a single oral dose of 200 mg artefenomel on day 10 (*vertical dashed line*).

For parasitemia at the time of dosing with 200 mg of artefenomel, parasite counts ranged from 30 331/mL to 175 522/mL (Figure 2 and Supplementary Figure 1). Initial clearance of parasitemia was observed in all participants; the regression models of the log-linear relationship of the parasite decay for all 8 participants were significant (*P* < .001) and contributed to the PD analysis of parasite clearance. The log_10_ PRR_48_ was 1.67 (95% confidence interval [CI], 1.55–1.78) and the corresponding parasite clearance half-life was 8.67 hours (8.11–9.31 hours). After the initial period of rapid parasite clearance occurring within 24 hours of artefenomel dosing, a second period of slower parasite clearance occurred over the course of 2–5 days, likely reflecting the clearance of gametocytes (see gametocytemia results below). Recrudescence was observed in 7 participants, 11–14 days after artefenomel administration, which was treated with artemether-lumefantrine (Supplementary Figure 1). The remaining participant received treatment with artemether-lumefantrine 18 days after artefenomel dosing.

### PK/PD Modeling

Artefenomel concentration-time profiles were adequately described by a 3-compartment disposition model with first-order absorption, a lag time, and linear elimination (Supplementary Table 4). A direct effect (*E*_max_) model best described the relationship between artefenomel plasma concentration and parasite killing rate (Supplementary Table 5). The PK/PD model estimated the median MIC of artefenomel to be 0.62 ng/mL (95% CI, .42–.76) and the median MPC_90_ to be 0.83 ng/mL (.55–1.05 ng/mL). Modeling simulations of various single doses of artfenomel in malaria patients estimated 300 mg to be the minimum effective dose needed to clear 10^9^ parasites per milliliter with 95% certainty. This dose was predicted to maintain artefenomel plasma concentrations above the MPC_90_ for a median of 11.8 days (95% CI, 3.8–23.1 days).

### Gametocytemia and Transmission to Mosquitoes

**Figure 3.**
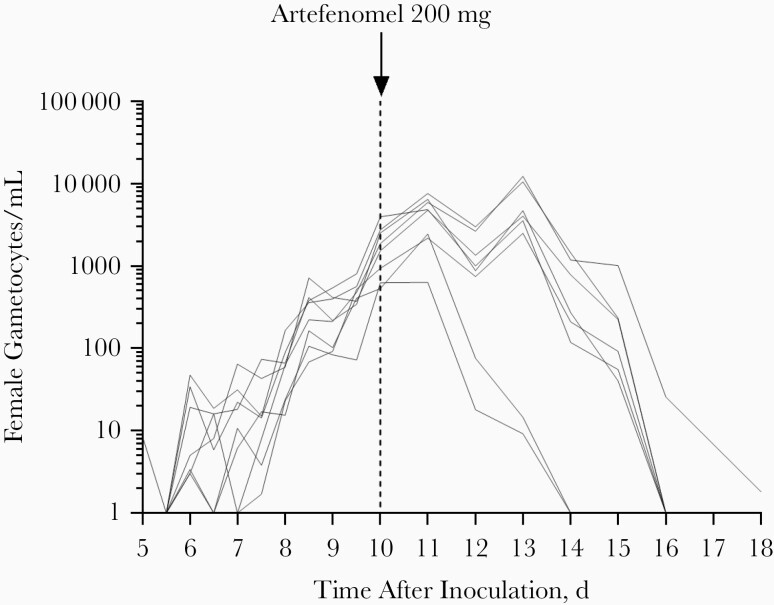
Individual participant gametocytemia profiles. Participants (n = 8) were inoculated intravenously with *Plasmodium vivax*–infected erythrocytes on day 0 and were administered a single oral dose of 200 mg artefenomel on day 10 (*vertical dashed line*).

The profile of gametocytemia development was similar to that of total parasitemia, steadily increasing until administration of artefenomel on day 10 (Figure 3 and Supplementary Figure 1). For gametocytemia at the time of dosing, female gametocyte counts ranged from 532/mL to 3951/mL. Gametocytes were cleared in all participants within 4–8 days of dosing, although the rate of gametocyte clearance seemed substantially slower than that of asexual parasites. A distinct lag phase of approximately 1–3 days was evident between the time of artefenomel administration and the time gametocytemia began to clear. Gametocytes reappeared after recrudescence of asexual parasitemia and were cleared with artemether-lumefantrine treatment (Supplementary Figure 1).

The transmission of gametocytes to *Anopheles* mosquitoes was evaluated with direct skin feeding and membrane feeding assays on day 8, and day 10 before artefenomel dosing. The mosquito feeding rates were high (mean, 95.2%; range, 76.9%–100.0%) and mortality rates were low (mean, 10.6%; range, 2.9%–40.6%), indicating good colony health (Supplementary Table 8). Transmission was achieved from 75% of participants (6 of 8) on day 10, but no transmission was observed on day 8 (Table 4). Mosquito infection rates ranged from 2% to 63% and were highest for the participant who had the highest level of gametocytemia at the time of feeding (gametocyte count, 3951/mL), with 63% of mosquitoes from the direct feeding assay and 12% from the direct membrane feeding assay becoming infected.

**Table 4.** Individual Participant Mosquito Transmission Results

Participant No.	Mosquito Infection Rate, Mosquitoes With Oocysts, No./Mosquitoes Tested, No (%)			
	d 8 After Inoculation		d 10 After Inoculation^a^	
	DFA	DMFA	DFA	DMFA
1	0/30 (0%)	0/50 (0)	0/19 (0)	0/50 (0)
2	0/29 (0)	0/50 (0)	0/29 (0)	1/50 (2.0)
3	0/30 (0)	0/50 (0)	0/30 (0)	0/50 (0)
4	0/30 (0)	0/50 (0)	1/30 (3.3)	NP
5	0/29 (0)	0/50 (0)	2/30 (6.7)	1/50 (2.0)
6	0/28 (0)	0/50 (0)	19/30 (63.3)	6/50 (12.0)
7	0/30 (0)	0/50 (0)	2/30 (6.7)	0/50 (0)
8	0/30 (0)	0/50 (0)	0/30 (0)	1/50 (2.0)

Abbreviations: DFA, direct feeding assay; DMFA, direct membrane feeding assay; NP, not performed.

^**a**^Mosquito transmission assays on day 10 were performed immediately before artefenomel dosing.

## DISCUSSION

The study aimed to investigate the safety, PK, and PD antimalarial activity of artefenomel when administered as a single oral dose of 200 mg to healthy malaria-naive participants experimentally infected with blood-stage *P. vivax*. Furthermore, it aimed to define the PK/PD relationship of artefenomel and establish the minimum effective dose required to achieve *P. vivax* parasite clearance.

Artefenomel was generally safe and well tolerated, with no serious AEs reported. AEs related to malaria infection were consistent with those reported for previous *P. vivax* IBSM studies [12, 13]. AEs considered possibly related to artefenomel included mild vomiting, moderate fatigue, mild myalgia, and mild or moderate headache. However, these AEs were also considered to be potentially related to malaria and thus were not exclusively considered drug related. A number of expected malaria-related safety laboratory abnormalities were identified and were not regarded as drug related. These included clinically significant decreases in lymphocyte counts and asymptomatic transient elevations in transaminases. Decreases in lymphocyte counts are known to be associated with malaria infection and have been previously reported in malaria volunteer infection studies [27]. Similarly, the occurrence of asymptomatic and transient elevated transaminases has been observed in previous IBSM studies [28] and during natural infection [29]. A detailed description and discussion of transaminase changes in *P. vivax* IBSM will be presented separately.

The good safety profile of artefenomel observed in this study is consistent with previous clinical trials [18, 19]. In the first-in-human study, 56 participants were administered artefenomel at doses between 50 and 1600 mg, and only 1 AE (headache) was considered related to artefenomel in the 200-mg dose group [19]. None of the 24 participants treated with 100–500 mg of artefenomel in a *P. falciparum* IBSM study experienced artefenomel-related AEs [18]. Furthermore, the phase 2 study involving 82 adult patients with acute, uncomplicated *P. falciparum* or *P. vivax* malaria who were treated with 200–1200 mg of artefenomel did not reveal any serious safety risks associated with artefenomel [20]. Together, the safety results obtained from the clinical studies performed to date indicate that artefenomel is generally safe and well tolerated when administered orally to humans.

The PK profile of artefenomel observed in this study was consistent with the results reported previously in both healthy participants and malaria patients [18–20]. A single 200-mg dose of artefenomel resulted in initial clearance of *P. vivax* parasitemia in all participants. PD analysis indicated a moderate rate of parasite clearance, with a log_10_ PRR_48_ of 1.67 and corresponding parasite clearance half-life of 8.67 hours. This was slower than the clearance rate observed for *P. falciparum* when tested at the equivalent dose in the IBSM model (log_10_ PRR_48_, 2.22; parasite clearance half-life, 6.51 hours) [18].

As expected, recrudescence occurred in most participants (7 of 8), allowing the PK/PD relationship between artefenomel plasma concentrations and *P. vivax* parasitemia to be determined. The estimated MIC of 0.62 ng/mL was considerably lower than the MIC for *P. falciparum* (4.1 ng/mL) calculated using the IBSM model [18], perhaps reflecting the significant biological differences between the 2 species. Modeling simulations estimated that minimum effective dose to clear 10^9^*P. vivax* parasites per milliliter was 300 mg. Single doses between 200 and 1200 mg were found to be equally effective in clearing both *P. falciparum* and *P. vivax* malaria in Thai patients [20]. However, in the Thai study, definitive treatment (mefloquine plus artesunate for *P. falciparum*; chloroquine plus primaquine for *P. vivax*) was given 36 hours after artefenomel dosing; thus, the potential for recrudescence was not evaluated.

The current study also enabled in vivo assessment of the gametocytocidal activity of a 200-mg dose of artefenomel, because all participants developed gametocytemia together with the development of asexual parasitemia. Gametocytes were cleared in all participants within 8 days of dosing, supporting previous data indicating that artefenomel has activity against sexual stages of *P. vivax* in malaria patients at doses of 800 and 1200 mg [20]. However, without a control group, or definitive knowledge of the lifespan of preformed *P. vivax* gametocytes in the circulation, it is not possible to be certain whether the slight delay in clearance of gametocytes compared with asexual parasites that was observed in the current study indicates that the dose tested is less effective, or that gametocytes disappeared from circulation at the normal rate. Gametocytocidal activity is a highly desirable property for new antimalarials to prevent parasite transmission to vector mosquitoes, important for progress toward malaria eradication.

An exploratory objective of the current study was to determine the potential for healthy participants infected with blood-stage *P. vivax* to transmit parasites to *Anopheles* mosquitoes. The ability of artefenomel to prevent transmission was not assessed. Transmission was successful from the majority of participants (75%) in feeding assays performed on day 10 before artefenomel administration, but was not observed in feeding assays performed on day 8. This is likely due to the considerably higher level of gametocytemia on day 10 compared with day 8 (gametocyte count, 532–3951/mL on day 10 vs 15–164/mL on day 8). Indeed the participant in which the prevalence of transmission was highest had the highest gametocytemia. These results further validate the reproducibility of a *P. vivax* transmission model that was recently developed using IBSM [14], and they demonstrate the capacity to evaluate the efficacy of transmission-blocking interventions.

In conclusion, the current study has demonstrated that a single 200-mg oral dose of artefenomel is safe when administered to healthy participants experimentally infected with *P. vivax* and exhibits antimalarial activity toward both asexual parasites and transmissible gametocytes. A single dose of 300 mg is predicted to clear parasitemia in patients with *P. vivax* malaria. Finally, we further validated the use of *P. vivax* IBSM as a model for evaluating transmission-blocking interventions.

## Supplementary Data

Supplementary materials are available at *The Journal of Infectious Diseases* online. Consisting of data provided by the authors to benefit the reader, the posted materials are not copyedited and are the sole responsibility of the authors, so questions or comments should be addressed to the corresponding author.

jiaa287_suppl_Supplementary_methods_and_results

## Notes

Presented in part: Annual Meeting of the Population Approach Group in Europe, Stockholm, Sweden, 11–14 June 2019 (abstract I-01); Seventh International Conference on *Plasmodium vivax* Research, Paris, France, 26–28 June 2019.

***Acknowledgments.*** We thank the volunteers who participated in the study; staff at Q-Pharm who conducted the trial, including Gem Mackenroth and Suzanne Elliott; Claire Wang and all the staff at QPID for quantitative polymerase chain reaction analysis; and Brett McWhinney at Pathology Queensland for plasma drug concentration measurements. From QIMR Berghofer, we thank Katharine Trenholme for preparation of inoculum; Silvana Sekuloski and Caroline Dobbin for project management; Matthew Adams, Melanie Rampton and Hayley Mitchell for assistance with mosquito rearing and transmission studies; and Adam Potter for manuscript preparation.

***Disclaimer.*** The funders had no role in the design, conduct or analysis of the study.

***Financial support.*** This work was supported by Medicines for Malaria Venture, which is funded by a number of donors, including US Aid, Bill and Melinda Gates Foundation, the UK Department for International Development, Norwegian Agency for Development Cooperation, Irish Aid, Newcrest Mining, Australian Aid, the Swiss Agency for Development and Co-operation; and Wellcome Trust. This work was also supported by the National Health and Medical Research Council of Australia (practitioner fellowship to J. S. M.).

***Potential conflicts of interest.*** N. G., S. C., and J. J. M., are employed by Medicines for Malaria Venture (MMV), which funded the study. J. S. M. received funding from MMV to perform the study. All other authors report no potential conflicts. All authors have submitted the ICMJE Form for Disclosure of Potential Conflicts of Interest. Conflicts that the editors consider relevant to the content of the manuscript have been disclosed.
