# Supplementary material for: Antimalarial Activity of Artefenomel Against Asexual Parasites and Transmissible Gametocytes During Experimental Blood-Stage Plasmodium vivax Infection
Source: J Infect Dis. 2020 Jun 1;225(6):1062–9. doi: 10.1093/infdis/jiaa287 (PMC8922009; doi:10.1093/infdis/jiaa287)
Supplement: jiaa287_suppl_Supplementary_methods_and_results [file jiaa287_suppl_supplementary_methods_and_results.pdf]

SUPPLEMENTARY METHODS AND RESULTS

Antimalarial activity of artefenomel against asexual parasites and transmissible gametocytes during experimental blood-stage *Plasmodium vivax* infection

Katharine A Collins, Azrin N. Abd-Rahman, Louise Marquart, Emma Ballard, Nathalie Gobeau, Paul Griffin, Stephan Chalon, Jörg J. Möhrle, James S. McCarthy

Contents

Inclusion and Exclusion Criteria for participant enrollment 2
Schedule of events 5
Table S1. Schedule of events 5
Pharmacokinetic/pharmacodynamic modelling methods and results 7
Table S2: Studies included in the PK and PD datasets 7
Table S3. Demographic characteristics of participants included in the PK and PD datasets 8
Table S4. Population parameter estimates of the final PK model 10
Table S5. Population parameter estimates of the final PD model 11
Individual participant parasitemia and gametocytemia results 12
Figure S1: Individual participant parasitemia and gametocytemia profiles 12
Safety Results 13
Table S6. Summary of adverse events reported during the study 13
Table S7. Malaria clinical score recorded for each participant at the time of artefenomel dosing and peak score recorded during the study 14
Malaria transmission results 15
Table S8. Results of direct feeding assays and direct membrane feeding assays 15
References 16

Inclusion and Exclusion Criteria for participant enrollment

Inclusion Criteria

Participants eligible for inclusion in this study must fulfil **all** of the following criteria:

Demography

I01. Adults (male and non-pregnant, non-lactating female) participants between 18 and 55 years of age, inclusive who do not live alone (from Day 0 until at least the end of the anti-malarial drug treatment) and will be contactable and available for the duration of the trial and follow up period (maximum of 6 weeks).
I02. Body weight, minimum 50.0 kg, body mass index between 18.0 and 32.0 kg/m^2, inclusive.

Health status

I03. Certified as healthy by a comprehensive clinical assessment (detailed medical history and complete physical examination).
I04. Normal vital signs after 5 minutes resting in supine position:
• 90 mmHg < systolic blood pressure (SBP) <140 mmHg,
• 50 mmHg < diastolic blood pressure (DBP) <90 mmHg,
• 40 bpm< heart rate (HR) <100 bpm.
I05. Normal standard 12-lead electrocardiogram (ECG) after 5 minutes resting in supine position, QTcF≤450 ms (males and females) with absence of second or third degree atrioventricular block or abnormal T wave morphology.
I06. Laboratory parameters within the normal range, unless the Investigator considers an abnormality to be clinically irrelevant for healthy participants enrolled in this clinical investigation. More specifically for serum creatinine, hepatic transaminase enzymes (aspartate aminotransferase, alanine aminotransferase), and total bilirubin (unless the Participant has documented Gilbert syndrome) should not exceed the acceptable range listed in Appendix 5 of the study protocol and haemoglobin must be equal or higher than the lower limit of the normal range.
I07. As there is the risk of adverse effects of the investigational drug (OZ439), and standard curative treatment (Riamet) in pregnancy, it is important that any participants involved in this study do not get pregnant or get their female partners pregnant. Female participants must be considered as women of not childbearing potential (WONCBP) to be eligible. WONCBP is defined as:
• Spontaneous amenorrhoea for at least 1 year or spontaneous amenorrhea for at least 6 months confirmed by an FSH result above the laboratory defined range for post-menopausal)
• or permanently sterilised (eg tubal occlusion, hysterectomy, bilateral salpingectomy)

Female participants with same sex partners (abstinence from penile-vaginal intercourse), are eligible when this is their preferred and usual lifestyle. These participants must be Rh positive if biologically fertile and not planning IVF within the required contraception period.
Male participants to be dosed with OZ439 must agree to use a double method of contraception including condom plus diaphragm or condom plus stable oral/transdermal/injectable hormonal contraceptive by female partner from at least 14 days prior to the time of the dose of the study drug through 96 days (14 weeks) after the last dose of OZ439. Abstinent male participants must agree to start a double method if they start a sexual relationship during the study and for up to 96 days (14 weeks) following the last dose of OZ439.

I08. All participants must be Duffy Blood group positive. Female participants of childbearing potential should be blood group Rh positive.

Regulations

I09. Having given written informed consent prior to undertaking any study-related procedure.

Exclusion criteria

Medical history and clinical status

E01. Any history of malaria or participation to a previous malaria challenge study
E02. Must not have travelled to or lived (>2 weeks) in a malaria-endemic country/area during the past 12 months or planned travel to a malaria-endemic country during the course of the study.
E03. Has evidence of increased cardiovascular disease risk (defined as >10%, 5 year risk when greater than 35 years of age) as determined by the Australian Absolute Cardiovascular Disease Risk Calculator (http://www.cvdcheck.org.au/).

Risk factors include sex, age, systolic blood pressure (mm/Hg), smoking status, total and HDL cholesterol (mmol/L), and reported diabetes status.
E04. History of splenectomy.
E05. Presence or history of drug hypersensitivity, or allergic disease diagnosed and treated by a physician or history of a severe allergic reaction, anaphylaxis or convulsions following any vaccination or infusion.
E06. Presence of current or suspected serious chronic diseases such as cardiac or autoimmune disease (HIV or other immunodeficiencies), insulin-dependent and non-insulin dependent diabetes, progressive neurological disease, severe malnutrition, acute or progressive hepatic disease, acute or progressive renal disease, psoriasis, rheumatoid arthritis, asthma, epilepsy or obsessive compulsive disorder
E07. History of malignancy of any organ system (other than localized basal cell carcinoma of the skin or *in situ* cervical cancer), treated or untreated, within 5 years of screening, regardless of whether there is evidence of local recurrence or metastases
E08. Participants with history of schizophrenia, bi-polar disease, or other severe (disabling) chronic psychiatric diagnosis including depression or receiving psychiatric drugs or who has been hospitalized within the past 5 years prior to enrollment for psychiatric illness, history of suicide attempt or confinement for danger to self or others.
E09. Frequent headaches and/or migraine, recurrent nausea, and/or vomiting (more than twice a month)
E10. Presence of acute infectious disease or fever (e.g., sub-lingual temperature ≥ 38.5°C) within the five days prior to inoculation with malaria parasites.
E11. Evidence of acute illness within the four weeks before trial prior to screening that the Investigator deems may compromise participant safety.
E12. Significant inter-current disease of any type, in particular liver, renal, cardiac, pulmonary, neurologic, rheumatologic, or autoimmune disease by history, physical examination, and/or laboratory studies including urinalysis.
E13. Participant has a clinically significant disease or any condition or disease that might affect drug absorption, distribution or excretion, e.g. gastrectomy, diarrhoea.
E14. Participation in any investigational product study within the 12 weeks preceding the study.
E15. Blood donation, any volume, within 1 month before inclusion or participation in any research study involving blood sampling (more than 450 mL/ unit of blood), or blood donation to Red Cross (or other) blood bank during the 8 weeks preceding the reference drug dose in the study.
E16. Participant unwilling to defer blood donations to the ARCBS for 6 months.
E17. Medical requirement for intravenous immunoglobulin or blood transfusions.
E18. Participant who has ever received a blood transfusion.
E19. Symptomatic postural hypotension at screening, irrespective of the decrease in blood pressure, or asymptomatic postural hypotension defined as a decrease in systolic blood pressure ≥20 mmHg within 2-3 minutes when changing from supine to standing position.
E20. History or presence of alcohol abuse (alcohol consumption more than 40 g per day) or drug habituation, or any prior intravenous usage of an illicit substance.
E21. Smoking more than 5 cigarettes or equivalent per day and unable to stop smoking for the duration of the study.
E22. Ingestion of any poppy seeds within the 24 hours prior to the screening blood test (participants will be advised by phone not to consume any poppy seeds in this time period).
E23. Excessive consumption of beverages containing xanthine bases, including Red Bull, chocolate etc. more than 400 mg caffeine per day (equivalent to more than 4 cups per day).

Interfering substance
E24. Any medication (including St John's Wort) within 14 days before inclusion or within 5 times the elimination half-life (whichever is longer) of the medication.
E25. Any vaccination within the last 28 days.
E26. Any corticosteroids, anti-inflammatory drugs, immunomodulators or anticoagulants. Any participant currently receiving or having previously received immunosuppressive therapy, including systemic steroids including adrenocorticotrophic hormone (ACTH) or inhaled steroids in dosages which are associated with hypothalamic-pituitary-adrenal axis suppression such as 1 mg/kg/day of prednisone or its equivalent or chronic use of inhaled high potency corticosteroids (budesonide 800 µg per day or fluticasone 750 µg).
E27. Any recent (< 6 weeks) or current systemic therapy with an antibiotic or drug with potential anti-malarial activity (i.e. chloroquine, piperaquine, benzodiazepine, flunarizine, fluoxetine, tetracycline, azithromycin, clindamycin, hydroxychloroquine, etc.)

General conditions
E28. Any participant who, in the judgment of the Investigator, is likely to be noncompliant during the study, or is unable to cooperate because of a language or mental deficit.
E29. Any participant in the exclusion period of a previous study according to applicable regulations.

E30. Any participant who lives alone (from Day 0 until at least the end of the anti-malarial drug treatment).
E31. Any participant who cannot be contacted in case of emergency for the duration of the trial and up to 2 weeks following end of study visit.
E32. Any participant who is the Investigator or any sub-investigator, research assistant, pharmacist, study coordinator, or other staff thereof, directly involved in conducting the study.
E33. Any participant without a good peripheral venous access.

Biological status
E34. Positive result on any of the following tests: hepatitis B surface (HBs Ag) antigen, anti-hepatitis B core antibodies (anti-HBc Ab), anti-hepatitis C virus (anti-HCV) antibodies, anti-human immunodeficiency virus 1 and 2 antibodies (anti-HIV1 and anti HIV2 Ab).
E35. Any drug listed in Table 2 of the study protocol (Drug Screening) in the urine drug screen unless there is an explanation acceptable to the medical investigator (e.g., the participant has stated in advance that they consumed a prescription or OTC product which contained the detected drug) and/or the Participant has a negative urine drug screen on retest by the pathology laboratory.

Specific to the study
E37. Cardiac/QT risk
• Family history of sudden death or of congenital prolongation of the QTc interval or known congenital prolongation of the QTc interval or any clinical condition known to prolong the QTc interval.
• History of symptomatic cardiac arrhythmias or with clinically relevant bradycardia. Electrolyte disturbances, particularly hypokalaemia, hypocalcaemia, or hypomagnesaemia.
• Electrocardiogram (ECG) abnormalities in the standard 12-lead ECG (at screening) which in the opinion of the Investigator is clinically relevant or will interfere with the ECG analyses on study

E38. Known hypersensitivity to OZ439, or any of its excipients or 4-aminoquinolines, artemether or other artemisinin derivatives, lumefantrine, or other arylaminoalcohols.
E39. Known severe reaction to mosquito bites other than local itching and redness.
E40. Unwillingness to abstain from consumption of citrus (grapefruit, Seville orange, etc.) for ≥21 days prior to initiation of the study (inoculation; Day 0) and for the study duration.
E41. Unwillingness to abstain from consumption of quinine containing foods/beverages such as tonic water, lemon bitter, from inoculation (Day 0) to the end of the malaria treatment.
E42. Any history or presence of lactose intolerance.
E43. Use of prescription drugs, herbal supplements, within four weeks prior to administration of the study drug, and/or over-the-counter (OTC) medication, dietary supplements (including vitamins) within two weeks prior to initial dosing. If needed (i.e. an incidental and limited need) paracetamol is acceptable up to 2 g/day.

Schedule of events

Table S1. Schedule of events

**Procedures**	**Screen**	**Challenge Inoculum *(P. vivax)***	**Malaria Monitoring**		**Mosquito Feeding Days**	**OZ439 (200mg) Drug Treatment**		**Rescue OZ439 (400mg) Drug Outpatient Treatment (if recrudescence)**		**Safety Monitoring**	**Riamet Treatment**	**Safety Monitoring**	**Final Visit or EOS**
**Day**	**-d28 to –d3**	**0**	**1,2& 3**	**4(AM) until PCR+ve for malaria**	**~ 1-3 days pre OZ439 confinement dosing**	**Admission**	**72h Confinement at clinical site (~study day 10)**	**Pre-dose**	**Treatment**	**Up to 21 days post OZ439 confinement dosing**	**Timing as outlined in the protocol**	**Study day (Riamet +24h, +48h)**	**Day 28±3**
Informed consent & eligibility	X												
Medical History	X												
Physical Examination	X	X		X		X	X	X	X	X	X	X	X
ECG	X	X				X	X	X	X		X		X
Vital Signs –	X	X		X		X	X	X	X	X	X	X	X
Haem & Biochem	X	X				X	X	X	X		X	X	X
LFT Monitoring#		X								X*			X
Serology & special tests	X	X											X
Pregnancy test/FSH	X	X				X		X					X
Red cell Allo-Antibody	X												X
Urinalysis	X												X
Urine Drug Screen	X	X				X							
Alcohol breath test	X	X				X		X					
**Blood stage challenge**		X											
**Phone Call**			X									X	
Clinical Score Assessment				X	X	X	X	X	X	X			
Unit Confinement						X	X	X	X				
**OZ439 Treatment**						X			X			X	
**Standard Treatment**											X	X	
**Adverse Event**		X	X	X		X	X	X	X	X	X	X	X
Malaria RT-PCR		X		X	X	X	X	X	X	X	X	X	X

OZ439 drug level (PK)						X	X	X	X	X			
Thick Blood Film					X								
Indirect Feeding Assay					X			X					
Direct Feeding					X								
Safety Serum Storage		X											X
Exploratory Bloods *(Optional)*		X			X	X				X			X
Breath Collection *(Optional)*		X		X	X	X	X			X	X	X	X
Glycocalyx Measurement *(Optional)*		X			X	X				X			X
Exploratory Urine collection *(Optional)*		X			X	X				X			X

*** 5 days post initial OZ439 dose**
# LFTs pre initial DFA and 5 days post initial DFA

Pharmacokinetic/pharmacodynamic modelling methods and results

Objectives

- To develop a population PK model of artefenomel in healthy participants inoculated with blood-stage *P. vivax* and *P. falciparum* malaria.
- To characterise the PK/PD relationship of artefenomel in healthy participants inoculated with blood-stage *P. vivax* malaria.
- To establish the minimum effective artefenomel dose required to achieve *P. vivax* parasite clearance from the blood of healthy participants.

Data

Data used for this analysis was taken from three phase 1 clinical trials (Table S2). The PK dataset derived from the current study (*P. vivax* IBSM study with single dose of artefenomel) as well as a previous IBSM study in which three doses of artefenomel were tested against *P. falciparum* using the IBSM model. The PD dataset derived from the current study as well as a previous *P. vivax* IBSM study in which participants were dosed with chloroquine (only pre-treatment data were used). The demographic characteristics of participants included in the PK and PD datasets are summarised in Table S3.

Table S2: Studies included in the PK and PD datasets

Study type (Trial identifier)	Artefenomel dose	No. of participants	Analysis dataset
P. vivax IBSM study (NCT02573857)	200 mg	8	PK and PD
P. falciparum IBSM study (ACTRN12612000814875)	100 mg	8	PK
	200 mg	8	
	500 mg	8	
P. vivax IBSM study (ACTRN12616000174482)	Not applicable (participants dosed with chloroquine)	24	PD (only data before drug administration)
Total		56	

Table S3. Demographic characteristics of participants included in the PK and PD datasets

Characteristic	Category	PK dataset (N=32)	PD dataset (N=32)
Gender	Female – n (%)	12 (37.5%)	11 (34.4%)
	Male – n (%)	20 (62.5%)	21 (65.6%)
Race	Asian – n (%)	0 (0%)	1 (3.1%)
	Indigenous Aboriginal – n (%)	0 (0%)	1 (3.1%)
	Latino – n (%)	0 (0%)	1 (3.1%)
	White – n (%)	32 (100%)	29 (90.7%)
Age (years)	Mean (SD)	25.3 (3.9)	25.5 (6.1)
	Range	20 – 34	19 – 44
BMI (kg/m^2)	Mean (SD)	23.5 (2.3)	23.4 (2.7)
	Range	18.8 – 28.2	18.8 – 29.5
Body Weight (kg)	Mean (SD)	74.1 (12.0)	74.2 (11.3)
	Range	53.9 – 97.6	57.2 – 99.5
Height (cm)	Mean (SD)	177.1 (9.5)	177.8 (9.7)
	Range	160 – 194	156 – 194

N: Number of participants, BMI: Body mass index, SD: standard deviation.

Methods

Software

The PK/PD modelling and simulation were conducted within R (v3.4.2) combined with the IQRtools package (v0.9.1) and Monolix (2016R1).

Population PK modelling

One-, two- and three-compartment models were considered to describe the concentration-time data. Different absorption and elimination models were also tested. Between subject variability (BSV) for each PK parameters was modelled using an exponential model. Additive, proportional and combined error models were tested to explain random unexplained variability (RUV). Covariates were selected on the basis of biological plausibility, statistical significance and clinical relevance. Age, sex, race, body weight, type of parasite and artfenomel dose were considered during covariate model building. Left censoring was used for concentrations below the lower limit of quantification (LLOQ, 1 µg/L).

Population PK/PD modelling

A sequential PK/PD modelling approach was performed in which the empirical Bayes estimates of individual PK parameters were used to predict artefenomel plasma concentrations at the same times when parasitemia was measured. The limit of detection (LOD) was 10 parasites/mL and was used for left-censoring. The geometric mean of biologic triplicate measurements of parasitemia by quantitative real-time polymerase chain reaction (qPCR) were natural log-transformed. To identify the relationship between artefenomel plasma concentration and parasite killing, several PD models were tested, such as direct effect (E_{max}), turnover, effect compartment and parasite clearance models. The BSV of each PD parameter was described using a log-normal variance model, with the exception of ln-transformed baseline parasitemia (PL_{base}), where normal distribution was assumed. An additive error model was used to present the RUV of the drug effect.

Simulation of PK/PD model

Simulations were undertaken to further assess the predictive performance of the final PK/PD model. The baseline parasitemia was randomly drawn for each patient from a previous phase 2a study in Thailand [1] involving *P. vivax* malaria patients (median: $10^{7.1}$ parasites/mL range: $10^{6.6}$ – $10^{7.7}$). In this phase 2a study, ten patients each were administered a single dose of 200, 400, 800 or 1200 mg artefenomel. Bayesian estimates of each patient's individual PK parameters were used to generate the individual concentration-time profiles. The parasitemia profiles of 500 trials of 10 participants in each dose group

were simulated. For each trial, the individual PD parameters were sampled from BSV distribution (normal distribution for PL_{base} and log-normal distribution for other PD parameters). The model simulated time courses for parasitemia at the median, 5th and 95th percentiles were constructed and overlaid with the observed parasitemia for each dose group.

Simulations were also performed to predict the minimum effective dose as defined as the dose that clears 10^9 parasites/mL. A total of 100 trials comprising 100 participants each were simulated for various doses in which population parameters were sampled from the uncertainty distribution and individual parameters were sampled from the BSV. The baseline parasitemia was randomly drawn for each patient from a previous phase 2a as described above. The growth rate was assumed to be 17.8-fold over a 48-hour erythrocytic cycle (0.06 h^{-1}), which was calculated from a previous *P. vivax* IBSM study (ACTRN12616000174482). Key efficacy parameters for each tested dose were determined.

Results

<u>Population PK modelling</u>

Population parameter estimates of the final PK model are listed in Table S4. Artefenomel concentration-time profiles were adequately described by a three-compartment disposition model with first-order absorption, a lag time and linear elimination following administration of single doses of 100, 200 and 500 mg. RUV was modelled using a proportional error model. Body weight was incorporated as an allometric function on clearance and volume of distribution parameters. Dose was found to correlate significantly with apparent clearance (CL/F). The estimated artefenomel CL/F was 85.7 L/h, with a BSV of 18.4%. Lower body weight and increased artefenomel dose resulted in lower CL/F.

<u>Population PK/PD modelling</u>

Population parameter estimates of the final PD model are listed in Table S5. An E_{max} model was used to describe the relationship of artefenomel plasma concentration on the parasite killing rate. The estimated maximum parasite killing rate (E_{max}) was 0.158 h^{-1} with a BSV of 21.7%. Individual estimates of efficacy parameters following administration of 200 mg artefenomel were: median log_{10} parasite reduction ratio over 48 hours ($log_{10}PRR_{48}$) of 2.0 (95% CI: 1.54 – 3.67), median parasite clearance half-life ($PCt_{½}$) of 7.23 h (95% CI: 3.94 – 9.4), median minimum inhibitory concentration (MIC) of 0.62 ng/mL (95% CI: 0.42 – 0.76), and median minimum parasiticidal concentration at which the parasite killing rate is 90% of its maximum (MPC_{90}) of 0.83 ng/mL (95% CI: 0.55 – 1.05).

<u>Simulation of PK/PD model</u>

The PK/PD model was externally validated by using it to predict the parasitemia-time profiles following single doses of 200, 400, 800 and 1200 mg in a phase 2a study. The model over predicted the parasitemia-time profiles at higher doses. Better prediction was observed when a higher E_{max} value (0.2 h^{-1}; derived from the *P. falciparum* IBSM study) was used in the simulation.

A 300 mg dose was predicted to be the minimum effective dose needed to clear 10^9 parasites/mL with 95% certainty. The median time above MIC (T>MIC) and median time above MPC_{90} (T>MPC_{90}) for a 300 mg single dose was predicted to be 14 (95% CI: 4.6 – 26.6) and 11.8 (95% CI: 3.8 – 23.1) days, respectively. The predicted median log_{10} PRR_{48} and median $PCt_{½}$ were 2.1 (95% CI: 0.8 – 4.2) and 6.9 h (95% CI: 3.4 – 18.9), respectively. *P. vivax* relapse and participant immunity are not taken into account in the simulations.

Table S4. Population parameter estimates of the final PK model

PARAMETER	VALUE	RSE	SHRINKAGE	COMMENT
F	1 (FIX)	-	-	Relative bioavailability (-)
CL/F	85.7	7	-	Apparent clearance (L/h)
V_c/F	215	23	-	Apparent volume of central compartment (L)
Q_1/F	7.59	12	-	Apparent inter-compartmental clearance between central and peripheral 1 compartments (L/h)
V_{p1}/F	1530	27	-	Apparent volume of peripheral 1 compartment (L)
Q_2/F	6.63	15	-	Apparent inter-compartmental clearance between central and peripheral 2 compartments (L/h)
V_{p2}/F	127	16	-	Apparent volume of peripheral 2 compartment (L)
K_a	0.297	20	-	Absorption rate constant (h^{-1})
T_{lag}	0.422	3	-	Absorption lag time (h)
**Inter-individual variability**				
ω_F	0 (FIX)	-	-	Log-Normal
$\omega_{CL/F}$	0.184	17	11%	Log-Normal
$\omega_{V_c/F}$	0.23 (FIX)	-	-	Log-Normal
$\omega_{Q_1/F}$	0.35	20	16%	Log-Normal
$\omega_{V_{p1}/F}$	0 (FIX)	-	-	Log-Normal
$\omega_{Q_2/F}$	0 (FIX)	-	-	Log-Normal
$\omega_{V_{p2}/F}$	0 (FIX)	-	-	Log-Normal
$\omega_{k_a/F}$	0 (FIX)	-	-	Log-Normal
$\omega_{T_{lag}/F}$	0.107	21	25%	Normal
**Parameter-Covariate relationship**				
$\beta_{CL/F,Dose}$	-0.438	16	-	Dose on CL/F
$\beta_{CL/F,WT0}$	0.75 (FIX)	-	-	Body weight on CL/F
$\beta_{V_c/F,WT0}$	1 (FIX)	-	-	Body weight on V_c/F
$\beta_{Q_1/F,WT0}$	0.75 (FIX)	-	-	Body weight on Q_1/F
$\beta_{V_{p1}/F,WT0}$	1 (FIX)	-	-	Body weight on V_{p1}/F
$\beta_{Q_2/F,WT0}$	0.75 (FIX)	-	-	Body weight on Q_2/F
$\beta_{V_{p2}/F,WT0}$	1 (FIX)	-	-	Body weight on V_{p2}/F
**Residual Variability**				
ε_{prop}	0.47	4	-	Compound concentration (µg/mL)

Table S5. Population parameter estimates of the final PD model

PARAMETER	VALUE	RSE	SHRINKAGE	COMMENT
PL_{base}	-2.42	10	-	Ln-transformed baseline parasitemia (parasites/mL)
k_{grow}	0·0536	2	-	Net parasite growth rate (h^{-1})
E_{max}	0.158	8	-	Maximum clearance rate (h^{-1})
EC_{50}	0.000645	10	-	Concentration achieving 50 % of maximum effect (µg/mL)
Hill	10 (FIX)	-	-	Hill coefficient (-)
**Inter-individual variability**				
$\omega_{PL_{base}}$	0.62	17	8.2	Normal
$\omega_{k_{grow}}$	0 (FIX)	-	-	Log-Normal
$\omega_{E_{max}}$	0.217	36	50	Log-Normal
$\omega_{EC_{50}}$	0.259	27	53	Log-Normal
ω_{Hill}	0 (FIX)	-	-	Normal
**Residual Variability**				
ε_{add}	0.992	3	-	Ln-transformed parasitemia (parasites/mL)

Conclusion

The PK and PD properties of artefenomel were characterised in healthy volunteers inoculated with blood stage *P. vivax*. Maximum killing rate was not achieved with 200 mg artefenomel. PK/PD modelling and simulation suggested that a minimum single dose of 300 mg is likely to clear 10^9 parasites/mL with 95% certainty.

Individual participant parasitemia and gametocytemia results

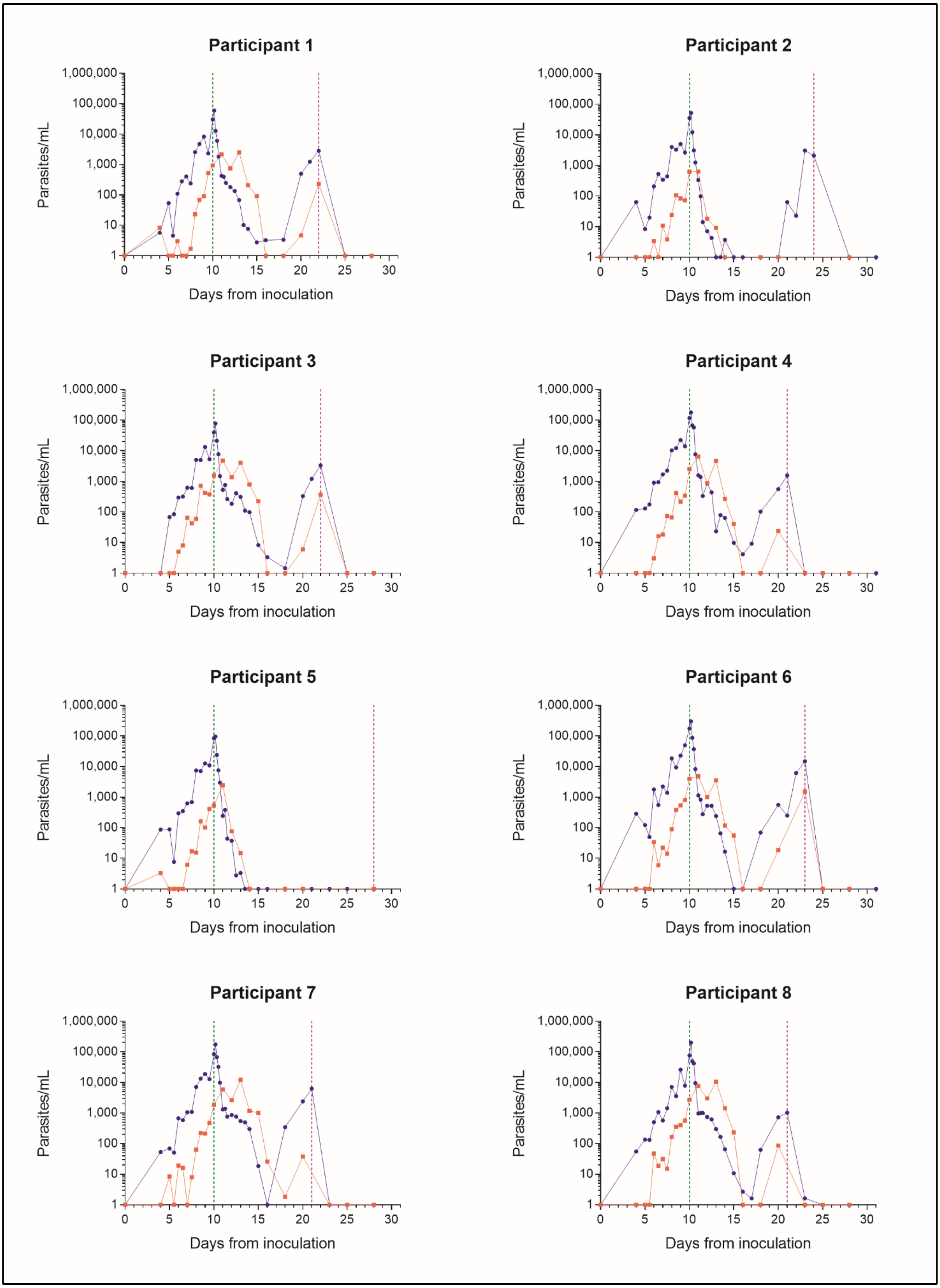

Figure S1: Individual participant parasitemia and gametocytemia profiles

Total parasitemia measured by qPCR targeting the gene encoding 18S rRNA is represented in blue. Gametocytemia measured by qRT-PCR targeting *pvs25* mRNA is represented in orange. Vertical green dashed lines represent time of treatment with OZ439. Vertical purple dashed lines represent time of treatment with artemether/lumefantrine.

Safety Results

Table S6. Summary of adverse events reported during the study

Adverse event	N participants	N events	Severity (N events)		
			Mild	Moderate	Severe
Abdominal discomfort	3	8	5	3	
Alanine aminotransferase increased	5	10		6	4
Anxiety	1	1		1	
Aspartate aminotransferase increased	5	5		4	1
Arthralgia	6	9	8	1	
Arthropod bite	4	6	6		
Chills	5	7	5	2	
Decreased appetite	3	3	3		
Diarrhoea	1	1		1	
Dizziness	1	1	1		
Fatigue	7	18	14	4	
Gingival swelling	1	1	1		
Headache	8	33	24	9	
Heart rate increased	1	1	1		
Hot flush	1	1	1		
Hyperhidrosis	4	5	5		
Influenza like illness	1	1	1		
Lethargy	1	1	1		
Lymphopenia	8	8		5	3
Malaise	6	8	7	1	
Muscle rigidity	2	2	2		
Myalgia	7	16	10	6	
Nausea	4	6	6		
Oropharyngeal pain	1	2	1	1	
Peripheral swelling	1	1	1		
Rash	1	1	1		
Pyrexia	8	30	16	11	3
Tachycardia	2	2	2		
Testicular disorder	2	2	2		
Upper respiratory tract infection	3	3	3		
Urticaria	1	1	1		
Vessel puncture site thrombosis	1	1	1		
Vomiting	1	1	1		
**TOTAL**		196	130	55	11

Table S7. Malaria clinical score recorded for each participant at the time of artefenomel dosing and peak score recorded during the study

Participant	Clinical score at time of artefenomel treatment (Day 10 AM)	Peak clinical score during study	Day of peak clinical score
1	10	10	Day 10 AM
2	3	7	Day 10 PM
3	2	6	Day 10 PM
4	3	6	Day 10 AM
5	2	2	Day 10 AM
6	13	14	Day 10 PM
7	0	13	Day 10 PM
8	11	11	Day 10 AM

The malaria clinical score served as a clinical indication of the severity of the induced malaria infection; 14 signs and symptoms commonly associated with malaria were graded using a 3-point scale (0=absent, 1=mild, 2=moderate, 3=severe) and the values were summed in order to generate an overall score (maximum possible score is 42).

Malaria transmission results

Table S8. Results of direct feeding assays and direct membrane feeding assays

Participant	Feeding rate (N mosquitos fed/N total mosquitos [%])		Mortality rate (N dead mosquitos/N total mosquitos [%])		Mosquito infection rate N mosquitoes with oocysts/N mosquitos tested (%)	
	DFA	DMFA	DFA	DMFA	DFA	DMFA
**Day 8**						
1	37/39 (94.9%)	73/73 (100.0%)	3/39 (7.7%)	7/73 (9.6%)	0/30 (0%)	0/50 (0%)
2	36/39 (92.3%)	70/71 (98.6%)	10/39 (25.6%)	3/71 (4.2%)	0/29 (0%)	0/50 (0%)
3	28/34 (82.4%)	68/72 (94.4%)	3/34 (8.8%)	6/72 (8.3%)	0/30 (0%)	0/50 (0%)
4	35/37 (94.6%)	61/70 (87.1%)	4/37 (10.8%)	9/70 (12.9%)	0/30 (0%)	0/50 (0%)
5	32/34 (94.1%)	61/66 (92.4%)	5/34 (14.7%)	3/66 (4.5%)	0/29 (0%)	0/50 (0%)
6	31/33 (93.9%)	50/65 (76.9%)	5/33 (15.2%)	6/65 (9.2%)	0/28 (0%)	0/50 (0%)
7	37/37 (100.0%)	61/64 (95.3%)	2/37 (5.4%)	6/64 (9.4%)	0/30 (0%)	0/50 (0%)
8	37/38 (97.4%)	62/66 (93.9%)	3/38 (7.9%)	3/66 (4.5%)	0/30 (0%)	0/50 (0%)
**Day 10**						
1	32/32 (100.0%)	79/81 (97.5%)	13/32 (40.6%)	9/81 (11.1%)	0/19 (0%)	0/50 (0%)
2	31/33 (93.9%)	70/73 (95.9%)	4/33 (12.1%)	8/73 (11.0%)	0/29 (0%)	1/50 (2.0%)
3	35/36 (97.2%)	77/77 (100.0%)	4/36 (11.1%)	6/77 (7.8%)	0/30 (0%)	0/50 (0%)
4	33/34 (97.0%)	NP	1/34 (2.9%)	NP	1/30 (3.3%)	NP
5	33/34 (97.0%)	75/77 (97.4%)	4/34 (11.8%)	4/77 (5.2%)	2/30 (6.7%)	1/50 (2.0%)
6	36/36 (100.0%)	74/75 (98.7%)	3/36 (8.3%)	20/75 (26.7%)	19/30 (63.3%)	6/50 (12.0%)
7	30/31 (96.8%)	64/67 (95.5%)	1/31 (3.2%)	6/67 (9.0%)	2/30 (6.7%)	0/50 (0%)
8	33/34 (97.0%)	61/62 (98.4%)	2/34 (5.9%)	2/62 (3.2%)	0/30 (0%)	1/50 (2.0%)

DFA- direct feeding assay; DMFA- direct membrane feeding assay; NP- not performed.

References

1. Phyo AP, Jittamala P, Nosten FH, Pukrittayakamee S, Imwong M, White NJ, et al. Antimalarial activity of artefenomel (OZ439), a novel synthetic antimalarial endoperoxide, in patients with *Plasmodium falciparum* and *Plasmodium vivax* malaria: an open-label phase 2 trial. Lancet Infect Dis. 2016;16: 61-69.
